# Development and validation of an objective virtual reality tool for assessing technical aptitude among potential candidates for surgical training

**DOI:** 10.1186/s12909-024-05228-1

**Published:** 2024-03-14

**Authors:** Noa Gazit, Gilad Ben-Gal, Ron Eliashar

**Affiliations:** 1 Department of Prosthodontics, Hadassah Medical Center, Faculty of Dental Medicine, Hebrew University of Jerusalem, Jerusalem, Israel; 2 Department of Otolaryngology/HNS, Hadassah Medical Center, Faculty of Medicine, Hebrew University of Jerusalem, Jerusalem, Israel

**Keywords:** Selection, Assessment, Surgical training, Technical skills, Aptitude, Validation

## Abstract

**Background:**

Good technical skills are crucial for surgeons. Yet although surgical training programs strive to assess technical aptitude when selecting surgical residents, valid assessments of such aptitude are still lacking. Surgical simulators have been proposed as a potentially effective tool for this purpose. The current study aims to develop a technical aptitude test using a virtual reality surgical simulator, and to validate its use for the selection of surgical residents.

**Methods:**

The study had three phases. In Phase 1, we developed an initial version of the technical aptitude test using the Lap-X-VR laparoscopic simulator. In Phases 2 and 3 we refined the test and collected empirical data to evaluate four main sources of validity evidence (content, response process, internal structure, and relationships with other variables), and to evaluate the feasibility and acceptability of the test. Specifically, Phase 2 comprised a review of the test by 30 senior surgeons, and in Phase 3 a revised version of the test was administered to 152 interns to determine its psychometric properties.

**Results:**

Both the surgeons and interns rated the test as highly relevant for selecting surgical residents. Analyses of the data obtained from the trial administration of the test supported the appropriateness of the score calculation process and showed good psychometric properties, including reliability (α = 0.83) and task discrimination (mean discrimination = 0.5, SD = 0.1). The correlations between test scores and background variables revealed significant correlations with gender, surgical simulator experience, and video game experience (*p*s < 0.001). These variables, however, explained together only 10% of the variance in test scores.

**Conclusions:**

We describe the systematic development of an innovative virtual reality test for assessing technical aptitude in candidates for surgical training, and present evidence for its validity, feasibility and acceptability. Further validation is required to support the application of the test for selection, as well as to discern the impact of gender, surgical simulator experience, and video game experience on the fairness of test results. However, the test appears to be a promising tool that may help training programs assess the suitability of candidates for surgical training.

**Supplementary Information:**

The online version contains supplementary material available at 10.1186/s12909-024-05228-1.

## Background

While many attributes are important for surgeons, currently, one quality in particular differentiates surgical specialties from all other medical specialties: the need for first-rate technical skills. Technical skills have been found to predict surgical outcomes, with superior technical proficiency associated with reduced complications, mortality, reoperations, and readmission rates even among experienced surgeons [1]. Thus, it is not surprising that studies defining the desired competencies of surgeons and surgical residents universally emphasize the importance of technical skills [2–7]. Moreover, technical skills have become if anything even more crucial in recent years, thanks to the growing popularity of minimally invasive surgery (MIS) in most surgical specialties. In particular, compared with traditional open surgery procedures, MIS requires significantly greater technical aptitude, such as hand-eye coordination, ambidexterity, visuospatial ability, and depth perception [8–10].

Technical aptitude varies greatly between individuals, likely reflecting both genetic and environmental factors [2, 9, 11–13]. Indeed, studies have found variability in technical abilities even among surgeons with extensive experience in their fields and high volumes of specific procedures performed [1, 12]. In addition, several studies have found that individuals acquire surgical skills at different rates, and that some (5–15%) never manage to achieve the expected level of competence despite extensive training [14–20]. Furthermore, Buckley et al. [21] found that the baseline technical aptitude of medical students was correlated with their rate of improvement in a laparoscopic simulator, and even their ability to improve at all. In their study, students with low baseline aptitude either achieved proficiency more slowly than those with high baseline aptitude, failed to reach proficiency despite slight improvement, or did not progress at all (30% of those students with low baseline aptitude). Taken together, the results of these studies, including the investigation by Buckley et al. [21], suggest that technical aptitude likely contributes to the variability in residents’ training outcomes. Therefore, training programs may benefit from assessing applicants’ technical aptitude, in addition to other relevant non-technical skills and characteristics [2–7], during their selection process.

Although assessing technical aptitude in candidates for surgical residencies may be beneficial to selection practices, it is not part of the current selection process in most programs worldwide. Rather, the selection process relies largely on tools and measures such as academic achievement, recommendations, and interviews [22, 23], which do not predict clinical or surgical performance during residency [24, 25]. While efforts have been made to identify tools for assessing technical aptitude, most of these have focused on surrogate tests, which serve as indirect indicators of non-specific technical abilities, usually in the traditional format of paper-and-pencil or computerized tests (e.g., the Mental Rotation Test, Pictorial Surface Orientation Test, or Purdue Pegboard Test). However, studies have shown inconsistent correlations between scores on such tests and surgical performance, and none of these tests have been shown to reliably predict trainee surgical performance [26, 27].

It has been proposed that surgical simulators might be more appropriate than surrogate tests for assessing candidates’ surgical aptitude since they designed to replicate real-life surgical tasks and, therefore, represent a job sample in which all relevant abilities can be assessed simultaneously [28, 29]. In recent decades, the majority of studies on surgical simulators have focused on validating these tools either for training [30] or for assessing the technical skills of surgeons and residents (e.g., for feedback, to measure progress during training, or as a means of examination or credentialing) [31, 32]. These studies have demonstrated that performance on virtual reality (VR) surgical simulators is correlated with performance in the operating room [28, 33, 34], that there is large variance in the performance and learning curves of trainees and surgeons in tasks performed on VR surgical simulators [14–20], and that these tasks are capable of effectively discriminating between surgeons of varying experience levels (beginners, intermediates, and experts) [35–37].

However, according to the contemporary framework of validity described by Messick [38, 39], this evidence for the validity of surgical simulators for assessing the proficiency of residents and surgeons cannot be applied to the possible use of these simulators for resident selection. According to this framework, validity is a characteristic of the use of an assessment tool for a specific purpose, and not a characteristic of the tool itself. It is therefore essential to validate the use of surgical simulators specifically for resident selection before incorporating them into these assessment procedures.

Although evidence validating the use of surgical simulators for resident selection is lacking, a number of studies, taking different perspectives, have provided initial evidence regarding the potential of using MIS simulators to assess candidates’ technical aptitude [8, 40–43]. Cope and Fenton-Lee [40] examined the performance of interns on six tasks using an MIS simulator. No differences were found between candidates who were interested in pursuing a surgical career and those who were not, suggesting a lack of self-selection. Jardin et al. [41] and Salgado et al. [43] found no significant correlation between tasks performed on a laparoscopic simulator and other available scores (USMLE scores, grades, interview scores). Therefore, they concluded that assessing candidates’ technical aptitude can improve the selection process. In a multi-method selection system developed by Gallagher et al. [8, 42], technical skills were assessed via four tasks performed on endoscopic and laparoscopic simulators, and some evidence of validity was assessed. However, the validity evidence they provided did not relate specifically to the technical skills assessment, but to the entire selection process, which included other types of assessments.

None of the aforementioned studies used a systematic process to develop a comprehensive test for assessing candidates’ technical aptitude based on accepted psychometric procedures (e.g., developing a test blueprint, systematic selection of tasks, developing a scoring system), or provided significant validity evidence for the use of surgical simulators in the selection process (i.e., test content, response process, internal structure, relationships to other variables, and consequences) [44]. In addition, most of these studies used MIS simulators to assess candidates for higher surgical education or surgical fellowships, so their assessment of technical skills is not applicable to candidates without previous surgical experience.

The current study addresses these gaps. In this research, we (1) systematically develop a VR simulation-based technical aptitude test for assessing candidates for surgical training, who have no previous surgical experience or knowledge; and (2) present initial evidence regarding its validity, feasibility, and acceptability for the selection of candidates for surgical training using the contemporary framework of validity [38, 39].

## Methods

The study had three main phases. In Phase 1, an initial version of a technical aptitude test using a laparoscopic simulator was developed. In Phase 2, expert surgeons reviewed the test and provided their feedback, which was used to revise the test. Finally, in Phase 3 the revised test was administered to a sample of interns. Based on these phases, we evaluated evidence for the validity, feasibility, and acceptability of the test for the purpose of selecting candidates for surgical training. Following the contemporary framework of validity [38, 39], we collected evidence from four sources: content, response process, internal structure, and relationships with other variables. Some of the evidence is based on the procedures used in the development and revision of the test (Phase 1), and some is based on the empirical data collected in Phase 2 and Phase 3. The study was approved by the ethics committee of the Hebrew University of Jerusalem, and all participants provided informed consent.

### Phase 1: test development

**Fig. 1:**
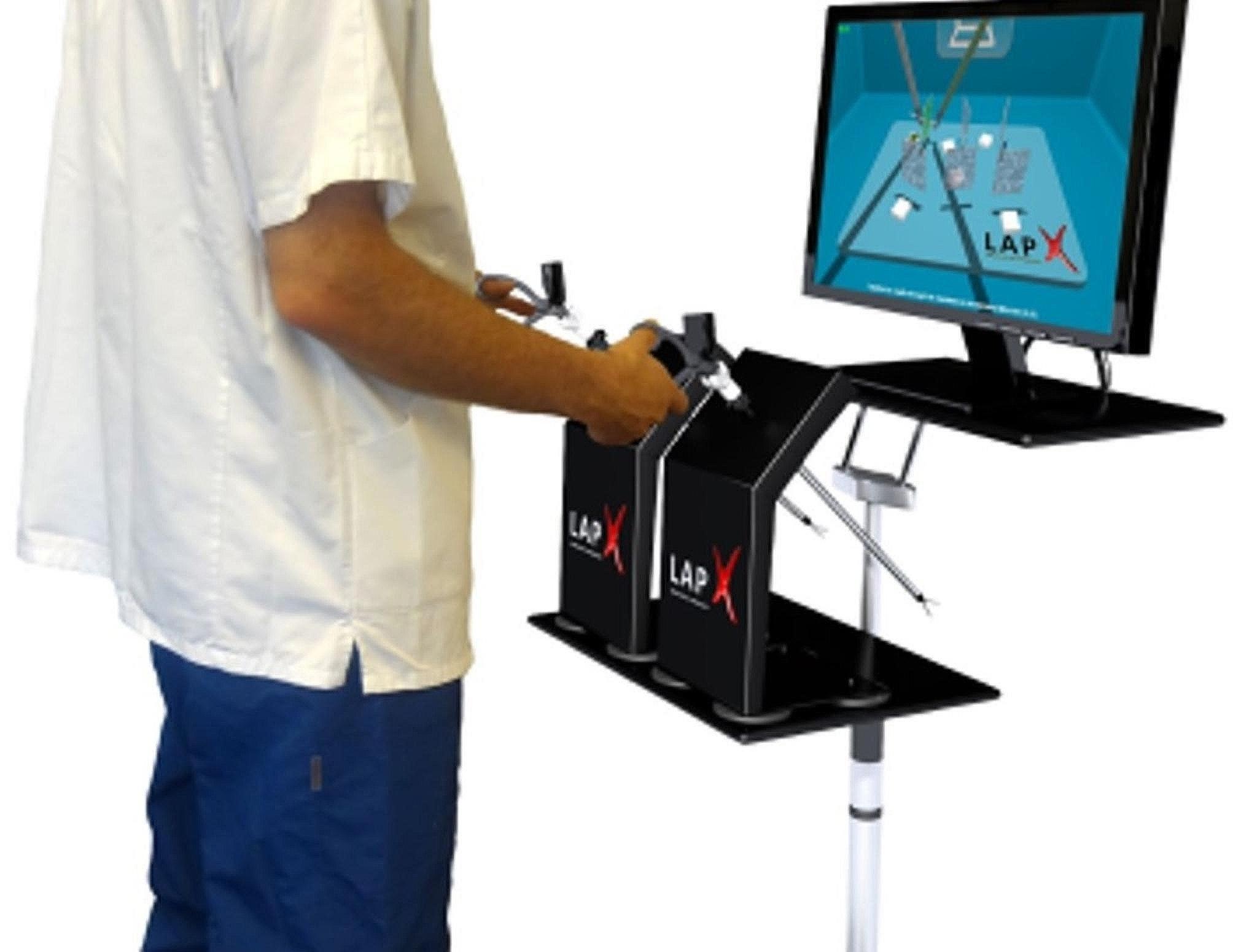
The Lap-X-VR laparoscopic simulator

The test was developed using the Lap-X-VR laparoscopic simulator (Medical-X, Netherlands; see Fig. 1), a computer-based virtual reality simulator validated for teaching basic laparoscopic skills [45]. The hardware includes two handles (controllers), one for the right hand and one for the left, which can be used to control three instruments: a mock grasper, scissors, or a camera (scope). To accommodate participants of different heights and ensure the comfort of the user, the simulator was placed on an adjustable desk.

We chose to use a VR laparoscopic simulator for the test development, rather than a basic box trainer, to ensure bias-free automatic assessment of participant performance. Specifically, we chose the Lap-X-VR simulator since it is highly portable, making it useful for testing purposes, and since its price is relatively affordable (~15,000 USD at the time of writing). However, to keep the simulator portable and low in cost, it does not provide haptic feedback.^1^

**Fig. 2:**
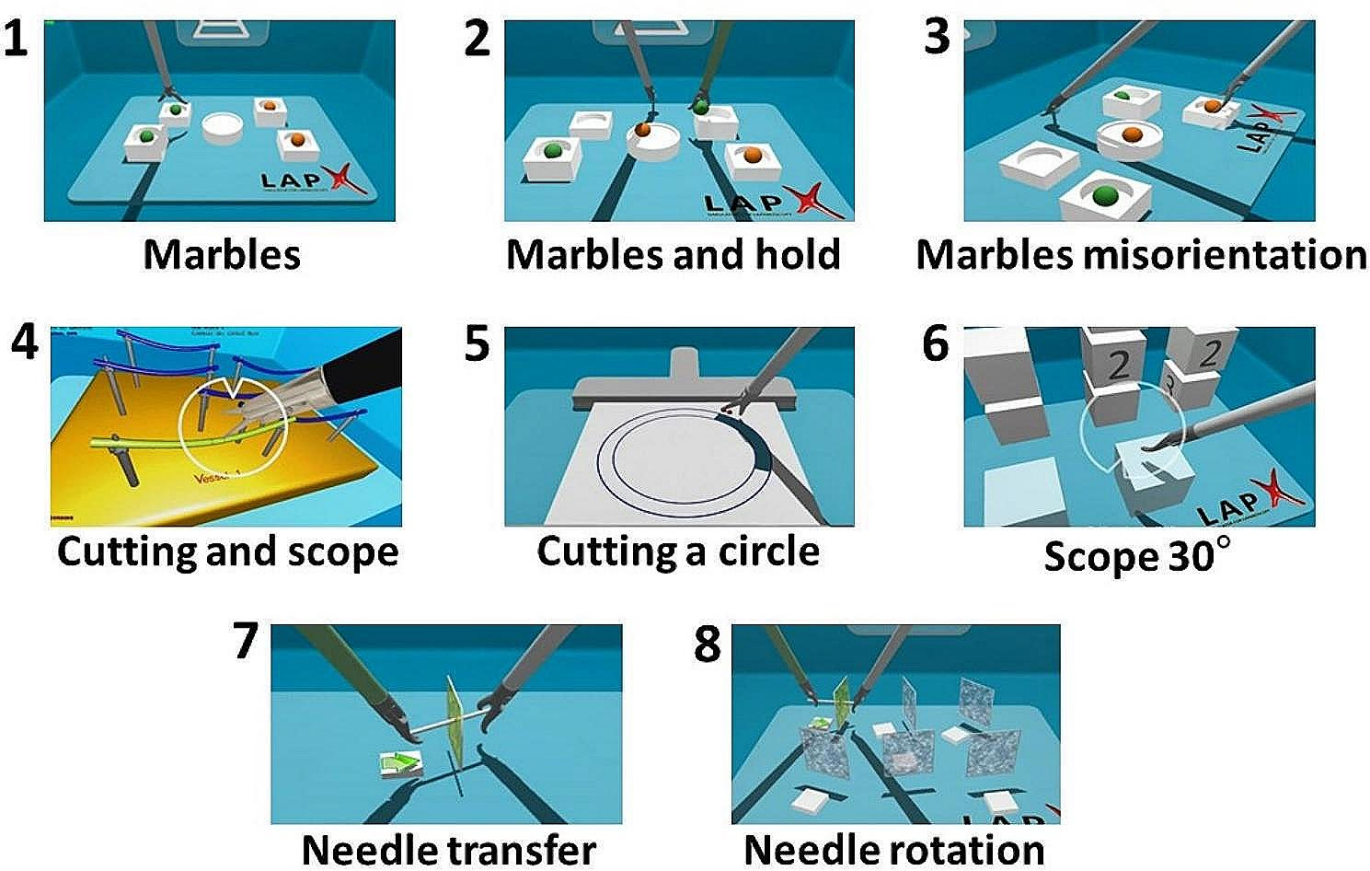
Illustrations of tasks selected for the initial version of the technical aptitude test. The tasks are ordered as they are in the test. The illustrations for Task 1, Task 2, and Task 4 show only the right-handed versions (i.e., Task 1a, Task 2a, and Task 4a, respectively). Task 8 appeared only in the initial version of the test and was omitted from later versions. The tasks are described in detail in Table A1 in the Appendix. The time limits set for the tasks were: Marbles (right/left) – 110 s, Marbles and hold (right/left) – 130 s, Marbles and hold misorientation – 150 s, Cutting and scope (right/left) – 90 s, Cutting a circle – 260 s, Scope 30° – 140 s, Needle transfer – 150 s, Needle rotation – 230 s

In developing the test, we first created an initial version following a blueprint developed by an educational assessment expert and three senior surgeons. This initial version of the test contained 11 basic skill tasks chosen from the 35 tasks available in the simulator’s software, all of which were designed according to the Fundamentals of Laparoscopic Surgery (FLS) curriculum. The 11 basic skill tasks we included were selected based on the expert blueprint to assess the perceptual and motor skills (coordination, ambidexterity, movement precision, visuospatial ability and depth perception) needed to perform all types of surgical tasks relevant to MIS (grasping and transferring objects; cutting with scissors; scope handling; suturing with a needle) [8, 10]. Procedural tasks which require previous surgical knowledge, or tasks that were considered too challenging for candidates without prior experience in laparoscopic surgery, were omitted. See Fig. 2 and Table A1 in the Appendix for illustrations and descriptions of the 11 selected tasks.

Within the test, the order of tasks was determined such that similar tasks appear in proximity (for example, the five marble tasks), and easier tasks appear before more difficult ones. Each task was performed once by each participant.^2^ However, in each task, participants were required to perform the same operation (mini-task) multiple times (4–10 times, depending on the specific task).

To ensure that the assessment would be objective and standardized, detailed instructions were written explaining how to use the simulator and how to perform each task, and a time limit was set for each of the tasks.^3^ Initially, the time limits were determined by doubling the time goal suggested by the simulator software for residents (i.e., the performance time that residents should achieve after practicing). The time limits were intended to ensure that most candidates would have sufficient time to complete the tasks despite not being familiar with them, and to allow ample variability in performance between different candidates (if time limits are too short, performance variability can be eliminated). The initial version of the test was then pilot-tested with eight medical students. Based on their feedback and the time it took them to complete the tasks, changes were made in the instructions, and time limits were adjusted if needed. The final time limits for each task are presented in Fig. 2 and in Table A1 in the Appendix.

To calculate the test scores we used performance data recorded by the simulator for the following parameters: success rate (%), time (sec), number of mistakes, path length (cm), and, where relevant, percent of time within scope (%). First, scores were calculated for each task separately. The task score calculations involved the following steps. First, since the performance parameters were measured using different scales, their raw values were transformed into z-scores (i.e., distributions with a mean of 0 and standard deviation of 1). Next, the scales for the time, number of mistakes, and path length parameters were reversed so that positive values represent better performance in the task (as for the success rate and percent of time within scope parameters).^4^ Then, a task score was calculated by averaging the z-score values (i.e., all parameters were given equal weight). Although some studies have reported computing total scores using weights determined by experts’ judgments (i.e., non-equal weights) [52, 53], we chose to compute the total composite scores based on equal weights^5^ since this method is more accurate and less biased [54, 55].

After computing the composite scores for each task, the total test score was calculated for each participant by averaging the scores for all tasks (again, with equal weights). To facilitate interpretation of the total test scores, they were transformed to a scale with a mean of 100 and standard deviation of 20.

### Phase 2: review by senior surgeons

#### Overview and participants

In Phase 2, the version of the test produced following the pilot with the medical students was reviewed by a sample of 30 experts (senior surgeons) from three hospitals in Israel. The 30 surgeons were specialists in one of five surgical fields selected for their extensive use of MIS techniques (general surgery, gynecology, orthopedics, otorhinolaryngology/head and neck surgery, and urology), and had at least 10 years of experience with MIS. To recruit participants, emails were sent to relevant surgeons asking for their participation. Email addresses of potential participants were obtained from hospital websites or from the database of the Israeli Medical Association (IMA).^6^ Recruitment continued until we had 30 participants, with at least two from each of the five surgical fields mentioned above. Surgeons who were willing to participate in the study were invited to review the technical aptitude test described above, and to share their opinions regarding the specific tasks and the whole test using a questionnaire.

#### Procedure and measures

First, participants received instructions and were allowed hands-on experience with each task included in the test. Following each experience, participants provided feedback by filling out the relevant section of the questionnaire. For each task, we elicited four responses: the relevance of the task for assessing technical aptitude in candidates for surgical training; the expected difficulty of the task for candidates; whether the time limit was appropriate; and whether the instructions were clear. The relevance and difficulty ratings were collected on 5-point Likert scales (1 = not relevant/very easy, 5 = extremely relevant/extremely difficult), and the time limit and instructions items were given as dichotomous yes/no answers. Upon completion of all tasks, participants were asked for three general evaluations: the relevance of the test as a whole in assessing the technical aptitude of candidates for surgical training; how comfortable it was working with the simulator; and how well the tasks simulated reality (based on their own prior experience with MIS and surgical simulators). All three general ratings were also collected on 5-point Likert scales, where 1 represents a low value and 5 a high value. Additionally, participants were invited to write comments and suggestions for improvement regarding each task and the whole test using an open format. At the end of the questionnaire, participants provided demographic information (age, gender, surgical specialty, and years of experience with MIS). The entire session took approximately 45 min to complete.

Based on the feedback provided by participants, the instructions for some tasks were slightly modified, and the needle rotation task, which was perceived as too challenging for candidates without surgical experience, was removed from the test (see under Results, below). The revised version of the test therefore included 10 tasks in total. In addition, based on the experts’ feedback, we added a 5-minute practice period before the test to reduce the effect of prior experience with surgical simulators.^7^

### Phase 3: trial administration to interns

#### Overview and participants

In Phase 3, the revised version of the test was administered to a sample of 152 medical interns from 10 hospitals in Israel. Medical interns were chosen for the study because interns in Israel are not selected based on technical skills, and so the characteristics of the general intern population should be similar to those of candidates for surgical training. To recruit participants, an invitation to participate in the study was posted in relevant Facebook and WhatsApp groups along with the contact information of the research coordinator. Recruitment continued until we had at least 150 participants. Participants received 35 USD, and were given feedback regarding their performance in the test relative to the rest of the sample (the percentile rankings of their total scores). The participants were invited one-by-one to complete the revised version of the test. They then filled out a feedback questionnaire.

It should be noted that the intern sample included (a) interns who were interested in pursuing a surgical career, (b) interns who were not interested in pursuing a surgical career, and (c) interns who had not yet decided. This composition allowed us to conduct additional analyses to test whether candidates self-select on the basis of their technical aptitude. Specifically, if interns do self-select based on their technical aptitude, those definitely interested in surgical careers should score better on our test than interns who do not plan to apply for surgical training, or who have not yet decided.

#### Procedure and measures

Participants first received instructions for taking the test and demonstrations of how to use the simulator. Instructions were given both orally by the research coordinator and in writing. Prior to starting the test, each participant was given five minutes for initial practice using the simulator.

In the test itself, the 10 tasks were presented successively. Each task was explained to participants in detail, including what was expected of them, what mistakes they should avoid, and how much time was allocated. Before performing each task, they also watched a short video, which demonstrated the ideal way to perform the task.

After completing all 10 tasks, participants were asked to provide feedback on each task and the whole test via a questionnaire. First, they were asked to complete three items for each task: the difficulty of the task (on a 5-point Likert scale, 1 = very easy, 5 = extremely difficult); whether the time limit was sufficient (yes/no); and whether the instructions were clear (yes/no). Following that, they were asked to provide two ratings for the test as a whole: its perceived relevance for selecting new surgical residents,^8^ and how comfortable it was working with the simulator (both on 5-point Likert scales, where 1 represents a low value and 5 a high value). As in Phase 2, participants were invited to write general comments and suggestions for improvement using an open format. Finally, participants provided general demographic information (age, gender, dominant hand, and desired training field: surgical/non-surgical). Participants also reported their previous experience using laparoscopic simulators^9^ and playing video games, both on 5-point Likert scales (1 = no experience, 5 = very extensive experience). The entire session took approximately 60 min to complete.

### Validation and analysis

Following the contemporary framework of validity [38, 39], we collected evidence from four sources: content, response process, internal structure, and relationships with other variables. We also collected evidence for the acceptability and feasibility of the test. The evidence is based both on the procedures used in the development and revision of the test (Phase 1), and on analysis of the empirical data collected in Phase 2 and Phase 3. For details on how we evaluated each source of validity, see Table 1.

**Table 1:** Summary of evidence collected in the study to assess the validity, feasibility, and acceptability of the test

Source of evidence	Definition	Relevant study phases^a^	Evidence collected in the study
Content	The degree to which the test content reflects the underlying construct it is intended to measure	Phase 1	Test development procedure designed to ensure adequacy of the test for assessment of technical aptitude (expert blueprint, pilot testing and revision, the use of simulated tasks)
		Phase 2	Relevance and difficulty ratings of expert surgeons Ratings of how well the tasks simulated reality General remarks or suggestion regarding the suitability of the test
Response process	The degree to which sources of error associated with test administration were eliminated	Phase 1	Test development procedure designed to minimize sources of error associated with test administration (detailed instructions, accommodation of the simulator to different needs of participants, allowing practice period for familiarization with the simulator)
		Phase 2 & Phase 3	Clarity of instructions ratings
	The appropriateness of combining different performance parameters into a composite score	Phase 3	Correlation between the different performance parameters (success rate, time, number of mistakes, path length, and percent of time within scope)
Internal structure	The quality of statistical and psychometric properties of the test	Phase 3	Item analysis (reliability, item discrimination)
Relationships with other variables	The degree to which the relationships of the test scores with other variables are consistent with the construct underlying the proposed interpretation of the test score	Phase 3	Correlations between the test scores and interns’ characteristics (age, gender, dominant hand, desired training, previous experience with surgical simulators, and previous experience with video games)
Feasibility	The practicality and ease with which a test or assessment can be given	Phase 2 & Phase 3	Assessment of the appropriateness of the time limits Assessment of the appropriateness of the instructions Assessment of how comfortable the use of the simulator is Difficulty ratings of specific tasks and for the test as a whole
Acceptability	The extent to which a test is viewed as suitable and appropriate by those who take it	Phase 3	Relevance ratings of interns

^a^ The study included three phases: Phase 1 (test development), Phase 2 (review of the test by expert surgeons) and Phase 3 (trial administration to interns)

The empirical data analyzed included the questionnaire data from both the expert surgeons and interns, and the performance data from the interns. The questionnaire data was analyzed by calculating descriptive statistics. The performance data from the test administration to interns was analyzed in three main steps. We first examined the distribution of each of the performance parameters in each task, and computed the Pearson correlations between the different parameters. Then, we calculated the test scores and conducted an item analysis to assess the reliability of the test and discrimination of each task. Finally, the correlations between the test scores and other background variables (age, gender, dominant hand, desired training field, previous experience with surgical simulators, and previous experience with video games) were assessed. For this purpose, we used *t*-tests to test for correlations with dominant hand, desired training field, and gender, and Spearman correlations for associations with age and the two previous experience variables. All statistical analyses were performed using R, version 4.2.2 (R Foundation for Statistical Computing, Vienna, Austria).

## Results

The demographic characteristics of the 30 expert surgeons who participated in Phase 2 and the 152 interns who participated in Phase 3 are presented in Tables 2 and 3, respectively. In addition, a summary of the questionnaire data from the expert surgeons and interns is presented in Table 4. In what follows, we present the results according to their relevance for the different sources of validity evidence and for feasibility and acceptability.

**Table 2:** Demographic characteristics of the senior surgeons (Phase 2)

Characteristic	Participants, *N* = 30; (%)
Average age in years (SD)	53.8 (8.4)
Female gender	4 (13)
Average years of experience with MIS (SD)	13.5 (7.9)
Surgical specialty	
General Surgery	8 (27)
Gynecology	5 (17)
Orthopedics	10 (33)
Otorhinolaryngology/Head and Neck Surgery	4 (13)
Urology	3 (10)

**Table 3:** Demographic characteristics of the interns (Phase 3)

Characteristic	Participants, *N* = 152; (%)
Average age in years (SD)	28.3 (3.8)
Female gender	71 (46)
Left dominant hand	13 (9)
Desired training field	
Surgical training	100 (65)
Non-surgical training	36 (24)
Not decided	17 (11)
Experience with MIS simulators	
No experience	18 (2)
Little experience	128 (12)
Moderate experience	54 (5)
Considerable experience	0 (0)
Very extensive experience	0 (0)
Experience with video games	
No experience	22 (14)
Little experience	45 (29)
Moderate experience	46 (30)
Considerable experience	20 (13)
Very extensive experience	20 (13)

**Table 4:** Relevance,^a^ difficulty,^b^ time limit,^c^ and clarity of instructions^d^ for each task in the test as rated by senior surgeons (Phase 2) and interns (Phase 3)

Task	Group	Relevance rating, mean (SD)	Difficulty rating, mean (SD)	Time limit, n (%)	Clarity of instructions, n (%)
Marbles (right or left hand)	Surgeons	3.6 (0.7)	2.7 (0.6)	29 (97)	29 (97)
	Interns	-	2.6 (0.6)	135 (88)	152 (99)
Marbles and hold (right or left hand)	Surgeons	3.9 (0.7)	3.6 (0.5)	26 (87)	30 (100)
	Interns	-	3.4 (0.7)	115 (75)	151 (99)
Marbles and hold – misorientation	Surgeons	3.5 (0.9)	4.4 (0.5)	24 (80)	30 (100)
	Interns	-	4.2 (0.6)	47 (31)	151 (99)
Cutting and scope (right or left hand)	Surgeons	3.8 (0.8)	3.2 (0.6)	27 (90)	30 (100)
	Interns	-	2.9 (0.7)	119 (78)	151 (99)
Cutting a circle	Surgeons	3.7 (0.8)	2.6 (0.6)	29 (97)	29 (97)
	Interns	-	2.8 (0.8)	126 (82)	143 (93)
Scope 30°	Surgeons	3.7 (0.7)	3.5 (0.6)	26 (87)	28 (93)
	Interns	-	3.3 (0.8)	131 (86)	149 (97)
Needle transfer	Surgeons	3.8 (0.6)	3.6 (0.5)	28 (93)	28 (93)
	Interns	-	3.3 (0.9)	86 (56)	147 (96)
Needle rotation^e^	Surgeons	2.6 (0.9)	4.7 (0.5)	20 (67)	29 (97)
	Interns	-	-	-	-

^a^ Only the expert surgeons were asked to the rate the relevance of the tasks. The relevance rating scale ranged from 1 to 5, with higher scores indicating greater relevance (1 – “not relevant”, 2 – “slightly relevant”, 3 – “moderately relevant”, 4 – “very relevant”, 5 – “extremely relevant”)

^b^ The difficulty rating scale ranged from 1 to 5, with higher scores indicating greater difficulty (1 – “very easy”, 2 – “easy”, 3 – “moderately difficult”, 4 – “very difficult”, 5 – “extremely difficult”)

^c^ Participants were asked whether the time limit was sufficient for the task. The number in the table represents the number of surgeons who responded “yes”

^d^ Participants were asked whether the instructions for the task were clear. The number in the table represents the number of surgeons who responded “yes”

^e^ This task was omitted from the test based on the expert surgeon’s feedback. Therefore, it was not included in the revised version administered to intern

### Content evidence

Content evidence of validity refers to the relationship between the test content and the construct it is intended to measure. To evaluate content evidence of validity, we assessed the feedback of the expert surgeons from Phase 2 regarding the relevance of the test, its difficulty, and the similarity of the tasks to reality (see Table 4). Examining the mean relevance and difficulty ratings for each unique task, it can be seen that the difficulty ratings varied between tasks, such that two tasks (the basic marbles task; cutting a circle) were perceived as having low to moderate difficulty, four tasks (marbles and hold; cutting and scope; scope 30°; needle transfer) were perceived as having moderate to high difficulty, and two tasks (marbles and hold – misorientation; needle rotation) were perceived as having very high difficulty. The mean difficulty rating across tasks was 3.5 (SD = 0.7), meaning that the test was perceived as difficult on average. Relevance scores were high (mean ratings of 3.5 or above) for seven of the eight unique tasks, with the needle rotation task being the exception. The mean relevance rating across tasks was 3.6 (SD = 0.4). There were no significant correlations between the relevance and difficulty ratings except in the needle rotation task, where we found a marginally significant negative correlation (*r*(28) = −0.3, *p* = 0.07). Since the needle rotation task was rated significantly less relevant than the other tasks, and also as extremely difficult for candidates without prior surgical experience,^10^ we decided to omit this task from the test. The revised version of the test therefore included 10 tasks (7 unique). The mean difficulty and relevance ratings of the tasks included in the revised version were 3.4 (SD = 0.6) and 3.7 (SD = 0.1) respectively.

Turning now to the whole-test ratings, the mean relevance rating of the whole test for selecting candidates for surgical training was high (M = 3.9, SD = 0.7). However, the degree to which performing the tasks simulated reality was rated only as moderate (M = 2.8, SD = 0.9). Based on the feedback we received from the surgeons, it appears that the relatively low results for similarity to reality stem from the lack of haptic feedback in our simulator system. Several experts also remarked that the presence of haptic feedback, such as that which exists in real laparoscopic surgeries, would also have increased their relevance ratings for the test. Indeed, there was a significant correlation between the whole-test relevance ratings and the similarity to reality ratings in the sample (*r*(28) = 0.4, *p* < 0.05). An additional concern raised by some of the surgeons was that the test includes only tasks relevant to MIS. According to them, to make the test even more relevant, it should include assessment of tasks relevant to open surgery, as well as tasks relevant to MIS.

In addition to the empirical evidence presented, some additional support for the content validity of the test comes from the procedures described above, which were used in the test development process: development of a blueprint by an expert committee based on job analysis, pilot testing and revision, selection of simulated tasks that mimic realistic surgical tasks, and using validated score parameters for performance [56]. These procedures were designed to ensure that the test developed would measure the relevant construct as accurately as possible.

### Response process evidence

Response process evidence has two components: first, showing that sources of error associated with test administration and understanding of instructions were eliminated; and second, determining the appropriateness of combining different performance parameters into a composite score.

To evaluate evidence for the first component, we analyzed the questionnaire data on the clarity of instructions and how comfortable it was using the simulator (see Table 4). Overall, both the expert surgeons and interns considered the instructions of the tasks to be appropriate, although some of the instructions were modified and improved further based on specific feedback provided by participants. These findings are in line with our efforts to create clear and detailed instructions for the test. In addition, the simulator platform was perceived as comfortable to use both by the expert surgeons (M = 3.4, SD = 0.8) and interns (M = 3.8, SD = 0.7). This is in line with the procedures performed to ensure comfort of use and to accommodate the simulator to different needs of participants.

Based on the written feedback, a relevant concern raised by the expert surgeons was that prior experience with surgical simulators could affect performance on the test. To minimize this effect and to ensure that participants understood how to use the simulator, we allowed examinees to interact with the simulator briefly before the start of the test (as described in the procedure of Phase 3). This practice period should reduce errors associated with misunderstanding how to use the simulator, and therefore should improve the validity of the test.

**Fig. 3:**
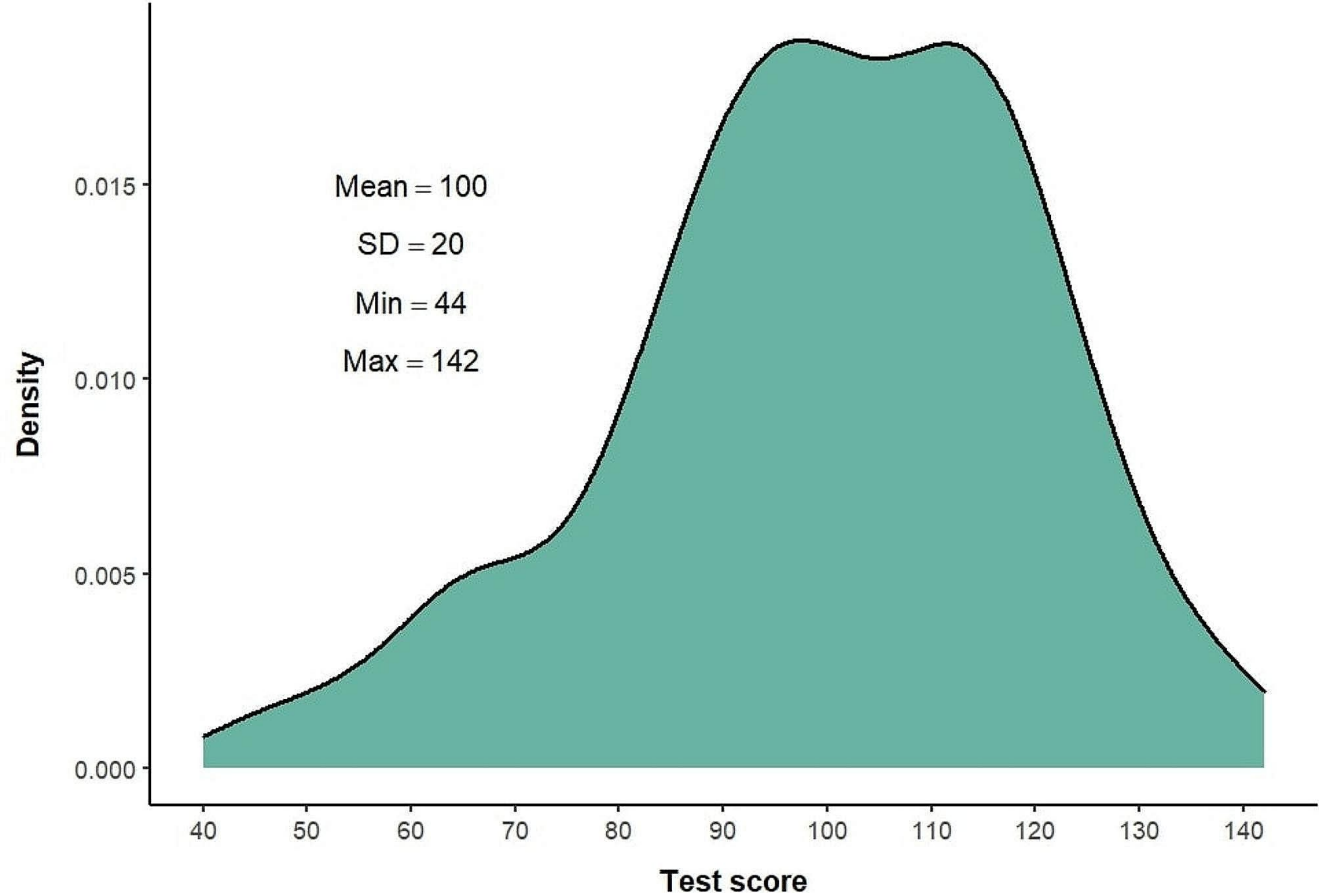
Distribution of the technical aptitude test scores among interns (*N* = 152)

To evaluate empirical evidence for the appropriateness of combining different performance parameters into a composite score, we analyzed the raw performance data of interns recorded for each task (success rate, time, number of mistakes, path length, and percent of time within scope). The means and standard deviations of each performance parameter for each task are shown in Table 5. Significant variability was found in each performance parameter in each task, eliminating the risk of a ceiling effect. The distribution of the five performance parameters varied between tasks in accordance with the difficulty of the task (see Figure A1 in the Appendix). To support the calculation of a composite score for each task based on the performance parameters, we examined the Pearson correlations between the parameters. The mean correlations between parameters across tasks are presented in Table 6 (see Table A2 in the Appendix for the correlations between parameters in each task). All correlations were statistically significant, supporting the calculation of a combined total score based on these parameters. Therefore, we calculated a total score for each task based on these parameters according to the procedure described in the Methods section. The total scores ranged from 44 to 142 (a range of 98). The distribution of the final test scores is presented in Fig. 3. It is evident that there is wide variation in the scores of different examinees, and that the distribution resembles a normal distribution.

**Table 5:** Descriptive statistics of the performance parameters (Phase 3)

	Mean (SD); *N* = 152					
Task	Success rate (%)	Time (sec)	Number of mistakes	Path length (cm)		Percent within scope (%)^a^
Marbles (right hand)	90.1 (20.6)	85.9 (20.2)	2.6 (3.9)	1398.1 (406.4)		-
Marbles (left hand)	95.1 (14.7)	76.1 (22.0)	2.8 (4.5)	1508.6 (533.1)		-
Marbles and hold (right hand)	82.6 (29.5)	100.3 (29.2)	8.5 (7.7)	2054.4 (749.3)		-
Marbles and hold (left hand)	77.6 (31.7)	103.5 (28.5)	12.3 (10.2)	2331.2 (881.2)		-
Marbles and hold – misorientation	52.1 (19.3)	148.2 (8.7)	13.1 (8.5)	3419.2 (998.0)		-
Cutting and scope (right hand)	84.0 (20.9)	82.4 (11.8)	2.8 (3.1)	1775.9 (442.4)		86.2 (13.0)
Cutting and scope (left hand)	90.3 (17.2)	77.9 (12.9)	4.3 (3.7)	2073.2 (586.2)		83.7 (15.2)
Cutting a circle	88.3 (12.9)	126.9 (22.8)	2.3 (2.4)	3516.9 (1214)		-
Scope 30°	93.3 (15.2)	219.6 (41.0)	9.7 (5.4)	1629.9 (717.8)		79.2 (14.2)
Needle transfer	63.9 (28.6)	145.1 (13.0)	1.9 (2.4)	3102.9 (855.8)		

^a^ The parameter of percent of time within scope was assessed only in tasks which require the use of a scope

**Table 6:** Correlations between performance parameters across tasks (Phase 3)

	Success rate	Time	Number of mistakes	Path length	Percent within scope
Success rate					
Time	-0.76^***^				
Number of mistakes	-0.50^***^	0.36^***^			
Path length	-0.39^***^	0.33^***^	0.68^***^		
Percent within scope	0.26^**^	-0.17^*^	-0.35^***^	-0.42^***^	

^*^p < 0.05, ^**^p < 0.01, ^***^p < 0.001

### Internal structure evidence

Internal structure, as a source of validity, relates to the statistical or psychometric properties of the test (e.g., test reliability and discrimination of test items). To assess evidence for the test’s internal structure we calculated the correlations between task scores, and conducted an item analysis based on the interns’ data from Phase 3.

Almost all the correlations between the task scores were high and significant (see Table A3 in the Appendix). According to the item analysis (see Table A4 in the Appendix), the test showed good psychometric properties: the discrimination of all 10 tasks was good (M = 0.5, SD = 0.1), and the test’s reliability (using the Cronbach’s alpha measure of internal consistency) was high (α = 0.83). The good discrimination indicates that the tasks differentiate between stronger and weaker interns based on their performance on the whole test, and the high correlations and internal consistency indicate that the tasks within the test measure the same characteristic or construct (i.e., technical aptitude). Therefore, the test seems to have good psychometric properties.

### Relationships with other variables

This source of evidence relates to the “degree to which these relationships are consistent with the construct underlying the proposed test score interpretation” [39]. Most commonly, this evidence is assessed based on correlations of assessment scores with a criterion measure, or with other tests measuring similar or different constructs (convergent and divergent evidence of validity, respectively). Such forms of evidence, though relevant for validation of the current test, were not available in this study. The present analysis relies on a different methodology, namely, examining whether the relationships found in this study between test scores and external variables are consistent with what is known from the literature regarding the relationship between technical aptitude and those variables. Based on the data of interns from Phase 3, we calculated the correlations between participants’ performance on the test and other variables: age, gender, dominant hand, desired training field (surgical or non-surgical), previous experience with surgical simulators, and previous experience with video games.

No significant correlations were found between scores on the test and either age, dominant hand, or desired training field. However, we found a significant correlation with gender, such that males (M = 105.0, SD = 16.5) obtained significantly higher scores than females (M = 94.1, SD = 22.1), with a mean difference of 10.9 (95% CI: 4.7–17.1), *t*(150) = 8.0, *p* < 0.001. The effect size, as measured by Cohen’s *d*, was 0.5, indicating a medium effect. To better understand this gender difference, we also compared the scores of males and females for each performance parameter separately (see Table A5 in the Appendix for the *t*-test statistics). Males scored higher than females in three performance parameters: success rate, time, and path length (*p*s < 0.01). However, no significant differences were found in the other two performance parameters (number of mistakes and percent within scope). In addition, we found weak but significant positive correlations between test scores and experience with either surgical simulators, *r*(150) = 0.19, *p* < 0.001, or video games, *r*(150) = 0.17, *p* < 0.001. Based on these findings, we conducted a hierarchical multiple regression to examine whether the correlation between gender and the test scores may be explained by different degrees of experience with surgical simulators or video games for males and females (see Table A6 in the Appendix for the regression statistics). Step one of the regression revealed that experience with either surgical simulators or video games contributed significantly to the model, *F*(2, 149) = 4.7, *p* < 0.05, and accounted for 6% of the variance in the total test scores. Introducing the gender variable (step 2) explained an additional 4.5% of the variance in test scores, and this change in *R*^2^ was significant, *F*(2, 148) = 7.5, *p* < 0.01. However, without accounting for experience with surgical simulators or video games, gender contributed 7.5% to the variance. Thus, the findings indicate that only some of the difference between genders can be attributed to different levels of experience with surgical simulators or video games, and that other factors may contribute to this gender difference.

In general, the correlations between scores on the technical aptitude test and the other examined variables are consistent with patterns found in the existing literature regarding the assessment of technical aptitude (we elaborate on this in the Discussion). Therefore, these findings provide evidence for the construct validity of the test.

### Feasibility and acceptability

We evaluated evidence for the feasibility and acceptability of the test, using data from the feedback questionnaires filled out by the expert surgeons and interns. Both the expert surgeons and interns considered the instructions for the tasks to be appropriate (as mentioned in the section on response process evidence above), and perceived the time limits as suitable. In addition, both groups perceived the simulator platform as comfortable to use (expert surgeons: M = 3.4, SD = 0.8; interns: M = 3.8, SD = 0.7). Although the difficulty ratings of surgeons and interns varied between tasks, the mean difficulty rating across tasks was medium (expert surgeons: M = 3.4, SD = 0.6; interns: M = 3.2, SD = 0.5), suggesting a reasonable level of difficulty. These findings support the feasibility of using the test for selection of candidates for surgical training. Finally, with respect to the acceptability metric, the interns found the test to be very relevant for selecting candidates for surgical training (M = 3.7, SD = 0.7), suggesting that the test is viewed as suitable (i.e., acceptable) by potential candidates.

## Summary and discussion

In this study, we present a systematic approach to the development of a VR technical aptitude test using a laparoscopic simulator, and provide initial evidence of its validity, feasibility and acceptability for resident selection. The evidence was assessed according to the contemporary framework of validity. The final version of the test takes approximately 50 min to complete and consists of 10 different tasks performed on the Lap-X-VR laparoscopic simulator.

Overall, the evidence presented supports the potential for using the test to select surgical residents. Here we address findings which require more elaboration, or which might be seen as threatening the validity of the test.

### The content of the technical aptitude test

Although the feedback from the experts in Phase 2 provided strong evidence for the relevance of the test’s content for assessing technical aptitude among candidates for surgical training, it also included two points that weaken somewhat the content evidence for validity. First, several surgeons pointed to the lack of haptic feedback in the simulator. We chose to forego haptic feedback in order to keep the simulator system portable and affordable, while recognizing the necessary trade-off between those considerations and the platform’s ability to simulate the full sensory experience of real-life scenarios. However, it should also be noted that haptic feedback is considered less important in the performance of basic tasks [46, 47] such as those used in the present test.

Second, some experts suggested enhancing the test’s relevance by incorporating tasks pertinent to open surgery. Indeed, we recognize that including tasks pertinent to open surgery as well as minimally invasive surgery would enhance the validity of the assessment. Nonetheless, we believe an assessment focusing solely on MIS-related tasks, such as that developed for this research, is still valuable, for several reasons. To begin with, MIS is becoming increasingly prevalent in many surgical fields, and therefore it is reasonable to give this method more weight in the assessment process. Second, MIS procedures require a greater level of technical skills compared to open procedures [8–10], and therefore they are significantly more difficult to learn [10, 57, 58]. In light of this, it is reasonable to emphasize MIS-related tasks when assessing candidates’ technical aptitude. However, although we consider the content evidence for validity in our current study to be substantial, we also acknowledge the potential contribution of broader evaluations that consider the full spectrum of surgical expertise. Future research should examine the impact of incorporating tasks associated with open surgery beyond MIS-related tasks on the selection of candidates for surgical training.

### Group differences in test scores

Our findings show no differences in test scores between interns who reported wishing to specialize in surgical fields vs. other medical fields. This finding is in keeping with previous studies showing that candidates do not self-select for surgical training based on their technical aptitude [40, 59–61]. In addition, this finding suggests that the exclusion of interns not interested in a surgical career would not affect the results of this study. Therefore, our sample of interns can be considered representative of the population of surgical training candidates in terms of their technical aptitude.

Furthermore, we found a small but significant relationship between test scores and participants’ previous experience with surgical simulators or with video games, with both of these variables explaining together 6% of the variance in total test scores. Specifically, the relationship between test scores and participants’ previous experience with surgical simulators was obtained despite the participants being permitted to practice for five minutes before the test, suggesting that a longer period of practice is required to further reduce the effect of previous experience with surgical simulators.

The findings that there is a significant correlation between test scores and participants’ previous experience with surgical simulators or video games are compatible with existing literature. Indeed, it is well-known that technical skills (such as FLS skills) can be improved significantly through practice and training with surgical simulators [17, 19, 62–64], and the literature also shows a consistent significant correlation between video game experience and novice performance on surgical simulators [26, 65–67]. On the face of it, this finding might cast doubt on the ability of our test to identify candidates with raw technical aptitude but no previous experience with either surgical simulators or video games, if candidates with greater experience were consistently more likely to score well. In our study, however, previous experience with surgical simulators or video games explained only 6% of the variance in test scores, indicating that such experience has little influence on scores in our test. In addition, it has been shown that although practice improves performance with surgical simulators, initial performance with the simulators predicts performance after practice [18, 19, 21]. Thus, even the slight advantage we observed in participants with prior experience can be countered through efforts to ensure that all candidates have the opportunity to train with simulators before the test. Under those conditions, the fact that technical skills can be improved with practice should not affect the ability of the test to distinguish candidates based on technical aptitude. Future work should encourage out-of-the-box thinking to expand opportunities for interns and medical students to experience and train with surgical simulators.

In addition, to differentiate between the effects of initial aptitude and training/learning on the development of technical skills and surgical outcomes, future studies should examine changes in test results during repeated exposure to surgical simulator training. Future studies should also examine whether longer practice periods (beyond the five minutes allotted in the current study) or the use of specific practice tasks can further decrease the effect of previous experience with surgical simulators or with video games on the test results.

Finally, we found a medium-sized difference between males’ and females’ technical aptitude scores, with males’ scores being significantly higher. This finding is in line with many previous findings of similar gender differences in visuospatial perception tasks [68–70] and in surgical simulator performance [19, 35, 71–76]. In some studies, these initial gender differences were eliminated after a period of practice [73, 77], but in other studies, the differences remained even after practice [74, 76, 78]. Historically, studies have suggested many factors that may explain these gender differences, of which some are related to “nature” (genetic and biological differences that affect brain functioning or morphology [79] and processing strategies [80]) and others to “nurture” (including different levels of exposure to activities involving spatial ability and coordination, such as video games and certain toys [81, 82]). Some studies have also suggested that test or situational characteristics, such as the design of surgical simulators [75, 76] or test characteristics that create “stereotype threats” [83], may contribute to some of these gender differences. In the present study, we believe the difference in scores we observed between males and females may be explained in part by the greater experience with simulators and video games among the males in our sample.

In addition, it is worth highlighting that we found gender differences in only three of the five performance parameters we measured, with males scoring higher on average in success rates, time, and path length, but not in number of errors or percentage of time within scope. This finding is in line with previous evidence showing that although male residents perform surgical tasks on a laparoscopic simulator faster than female residents, the genders do not differ in terms of number of mistakes [71]. Furthermore, a recent study focusing on cholecystectomy found that male surgeons performing the procedure were faster, but had less favorable outcomes in terms of surgical complications than female surgeons [84]. Considering these findings, it is possible that males and females tend to exhibit different profiles of performance characteristics, and that the weight assigned to each characteristic may result in different patterns of technical skill assessment scores between males and females.

Looking more closely at the specific implications of this gender differences for stakeholders – what is sometimes called consequential validity – we should note the potential implications of the correlations between test scores and gender. Although it is unclear to what extent these gender differences reflect deep-rooted differences in technical aptitude between men and women (whether due to nature or nurture), in practice it seems likely that selection based on any simulator-based technical aptitude test would favor men. Future research should examine whether the validity of performance parameters which favor males (e.g., time) differs from the validity of performance parameters which do not favor males (e.g., number of mistakes) for assessment of technical aptitude, and whether interventions aimed at leveling the playing field, e.g. by increasing women’s access to training on laparoscopic simulators or video games, would help to minimize differences in outcomes between the genders. Future research should also examine whether factors irrelevant to technical aptitude itself, such as the design of the simulator or the size of the handles, may help explain the performance differences found between men and women. Until these issues are resolved, surgical training programs might consider using different cutoffs or norms for men and women to avoid worsening the underrepresentation of women in surgery. Finally, it is important not to rely solely on a technical aptitude test in selecting candidates, but to include in the selection process assessments of non-technical skills, where women have no disadvantage relative to men, and may in fact tend to score higher [85, 86].

### Implications and consequences

Currently, most surgical programs worldwide do not employ a structured selection process for selecting surgical trainees based on technical aptitude or other non-technical characteristics. Some surgical programs (e.g., in the US, Canada, the UK, Ireland, Sweden, Denmark, the Netherlands, Australia, New Zealand, and Hong Kong) use some form of structured assessment process, but mostly to assess cognitive and personality factors [22, 87, 88]. A few programs in the UK, Ireland, Australia, and New Zealand include some form of technical skill assessment for candidates with prior surgical experience [9, 87, 88]. There is, however, no documented use of structured technical aptitude assessments for candidates without previous surgical experience. We argue that a primary reason most surgical programs worldwide do not assess candidates’ technical aptitude is the lack of objective and validated tools for this purpose. In this study, we implemented a systematic process to develop and validate a VR technical aptitude assessment test using a laparoscopic simulator. In light of the evidence presented regarding the feasibility, acceptability, and validity of the test, surgical programs may consider incorporating this test into their selection process, thus expanding the scope of the abilities evaluated.

Assessment of technical aptitude can help program directors identify the most talented candidates – those able to learn technical skills more easily, and at a faster rate. This is especially important today given that modern surgical methods such as MIS and robotic surgery require greater technical skills than before, and in light of other challenges such as work hour restrictions and economic pressure to improve efficiency in the operating room [22]. Equally, surgical programs can use the test not just to select candidates from the upper end of the distribution, but to screen out candidates from the lower end of the distribution. Candidates with low technical aptitude can then be directed to other medical specialties. This is essential since evidence demonstrates that even with continued practice, not all surgical trainees will achieve surgical competence (i.e., the ability to perform surgical procedures safely and independently) by the end of training [14–20, 64]. Improved selection may reduce the number of surgeons who complete their training program but are unable to operate to the required level of proficiency, and thereby are likely to increase patient safety. Future studies should examine the effect of different cut-off scores on the validity of the test for resident selection. Moreover, there is evidence not only that candidates do not currently self-select based on technical aptitude [40, 60, 61], but also that residents who feel that their operative skills are insufficient are more likely to drop out of residency [89]. Thus, the addition of technical aptitude to the selection process can help potential candidates assess their own suitability for surgical practice, allowing them to make informed decisions regarding their career path. Improved self-selection combined with a more informed selection process for surgical training may result in a better match between applicants and programs, and a reduction in resident attrition.

Finally, it is important to adapt the candidate selection process to changing demands in the field, as surgical methods and technology are constantly developing and improving. Future technologies may compensate for a lack of technical aptitude among surgeons. The introduction of 3D laparoscopic systems, for example, is expected to facilitate the performance of MIS procedures, and therefore to reduce the need for high visuospatial ability and hand-eye coordination among surgeons [90]. Changes such as these will result in lowering the importance placed on assessment of technical aptitude during selection for surgical training.

### Strengths and limitations

Key strengths of this study include its use of a systematic procedure to develop a test for assessing technical aptitude, based on accepted psychometric procedures, and the breadth of the evidence provided for all sources of validity. Another strength of the study is the large sample of expert surgeons and interns from various hospitals.

The study also has limitations. First, while our sample was large, both the experts and interns all came from one country, thereby limiting the generalizability of the results. Nonetheless, since technical aptitude should be distributed similarly across candidates from different countries, we can expect similar results in other countries. Second, since the interns in our study were volunteers, it is possible that our sample does not fully represent the population of candidates for surgical training. However, based on the large variability in test scores obtained in this study, and the similarity between our results and those of previous studies, we believe our sample is likely sufficiently representative in terms of the construct of interest (technical aptitude). Third, the interns in our study were paid for their participation, and it is possible that this may have influenced their responses to the feedback questionnaire. However, since participants completed the feedback questionnaire anonymously and were encouraged to provide honest feedback, we do not believe that this influence was significant. In addition, although this study presented various sources of evidence for the validity of the test, future studies should gather further evidence in areas which were beyond the scope of the current research, such as relationships between the test scores and performance criteria; comparisons between experts and novices; and comparison between the present test and other instruments (e.g., surrogate tests for assessment of dexterity or visuospatial ability). Finally, this study focused on assessing technical aptitude among candidates for surgical training, although other non-technical characteristics may also be important. To fully assess the suitability of candidates for surgical training, the selection process should include objective assessments of cognitive abilities and personality characteristics as well as technical aptitude [2–7]. Future studies should evaluate different methods for assessing non-technical skills in candidates for surgical training, and the validity of combining these evaluations with technical aptitude assessments.

## Conclusions

The present study presents the systematic development of a new technical aptitude test performed on the Lap-X-VR laparoscopic simulator, and provides initial evidence of its validity, feasibility, and acceptability. The test consists of 10 different tasks and takes approximately 50 min to complete. The evidence suggests that use of the test in selecting surgical residents may benefit training programs, patients, and in most cases trainees themselves. Further evidence is needed to support the validity of the test for selection of surgical residents and to discern the impact of gender, surgical simulator experience, and video game experience on the fairness of test results.

### Electronic supplementary material

Below is the link to the electronic supplementary material.

Supplementary Material 1

## Data Availability

The datasets used and/or analyzed during the current study are available from the corresponding author on reasonable request.

## Footnotes

1 Based on evidence showing that simulators which do not incorporate haptic feedback are not inferior to high-fidelity simulators with haptic feedback in terms of skill training and assessment (especially in the case of basic surgical tasks) [46–48], we assumed that this characteristic of the simulator would not have a significant impact on the validity of the assessment.

2 Our decision to present each task just once allowed us to include a variety of different tasks in the test, and thereby to assess different abilities relevant to technical aptitude, without making the full test overly time-consuming. This decision was based on ample evidence showing that although practice improves subjects’ performance, initial performance with surgical simulators predicts performance after a practice period (subjects with lower initial abilities require more time to learn and perform worse at the end) [14, 18, 20, 21, 49–51].

3 Time limits were used in order to facilitate administration of the test in the context of candidates for surgical training who do not have previous surgical experience and, therefore, may not be able to complete all the tasks in the test. When time limits are not set for each task, participants may continue to attempt the task for an infinite period of time, making the test less feasible to administer.

4 As percent of time within scope was measured only in those tasks which involved working with a scope, it was included in the total performance score only for those tasks.

5 We also examined the option of calculating total scores for each task while assigning double weights to success rate and number of mistakes compared to the other parameters, similar to Chowriappa et al. [52]. The correlations between these scores and the equal weight scores used in this study were very high (~0.98).

6 The IMA is the professional association representing about 95% of the physicians in Israel. The IMA represents physicians in all fields and regions throughout the country and at all stages of their medical careers.

7 As we could not determine the optimal length of the practice period in advance and did not wish to significantly prolong the test administration, we initially set the practice period at five minutes.

8 Participants were asked to answer this question based on their personal opinion to assess the acceptability of the test among potential candidates for surgical training.

9 A prior experience with simulation of motor skills that have not been performed on laparoscopic simulators was not assessed. However, it is unlikely that the participants, who were interns, had experience with motor skill simulations (in Israel, simulations of motor skills are not common before training).

10 In addition, as some of the experts pointed out, the fact that this task is too difficult for candidates without surgical training inherently makes it less relevant for selection.
