# Supplementary material for: Development and validation of an objective virtual reality tool for assessing technical aptitude among potential candidates for surgical training
Source: BMC Med Educ. 2024 Mar 14;24:286. doi: 10.1186/s12909-024-05228-1 (PMC10941473; doi:10.1186/s12909-024-05228-1)
Supplement: Supplementary file 1 — Supplementary Material 1 [file 12909_2024_5228_MOESM1_ESM.docx]

**Appendix**

**Table A1**. Description of the Tasks Included in the Technical Aptitude Test

**Figure A1**. Distributions of Performance Parameters in the Revised Test, by Task (Phase 3)

**Table A2**. Correlations Between Performance Parameters in Each Task (Phase 3)

**Table A3**. Correlations Between Task Scores (Phase 3)

**Table A4**. Item Analysis (Phase 3)

**Table A5**. Summary of Paired t-tests Analyses Comparing Males and Females' Parameter Scores (Phase 3)

**Table A6**. Summary of the Hierarchal Regression Analysis (Phase 3)

**Table A1**. Description of the Tasks Included in the Technical Aptitude Test

| Task | Task description | Surgical skills assessed | Initial time limit |
| --- | --- | --- | --- |
| 1a. Marbles (right hand) | The goal is to move the orange marbles from the right to the left, and the green marbles from the left to the right, using the right-hand instrument. | Grasping and transferring objects | 110 sec. |
| 1b. Marbles (left hand) | The goal is to move the orange marbles from the right to the left, and the green marbles from the left to the right, using the left-hand instrument. | Grasping and transferring objects | 110 sec. |
| 2a. Marbles and hold (right hand) | The goal is to move the orange marbles from the right to the left, and the green marbles from the left to the right, using the right-hand instrument (as in Task 1), while holding the left-hand instrument steady on the blue antenna located in the center of the screen. | Grasping and transferring objects | 130 sec. |
| 2b. Marbles and hold (left hand) | The goal is to move the orange marbles from the right to the left, and the green marbles from the left to the right, using the left-hand instrument (as in Task 2), while holding the right-hand instrument steady on the blue antenna located in the center of the screen. | Grasping and transferring objects | 130 sec. |
| 3. Marbles and hold – misorientation | The goal is to move the orange marbles from the right to the left, and the green marbles from the left to the right, using either of the instruments (right or left according to the examinee’s choice), while holding the other instrument steady on the blue antenna located in the center of the screen. After every successful transfer of a marble, the viewing angle changes, making the task more difficult as it progresses. | Grasping and transferring objects | 150 sec. |
| 4a. Cutting and scope (right hand) | This task features a network of six blue wires. One at a time, one of the blue wires will turn green. The goal is to cut the green wire between two demarcated lines using scissors controlled by the right-hand instrument. The scope is controlled by the left-hand instrument, and the scissors must stay within the scope’s field of vision as much as possible. | Cutting, scope handling | 90 sec. |
| 4b. Cutting and scope (left hand) | This task features a network of six blue wires. One at a time, one of the blue wires will turn green. The goal is to cut the green wire between two demarcated lines using scissors controlled by the left-hand instrument. The scope is controlled by the right-hand instrument, and the scissors must stay within the scope’s field of vision as much as possible. | Cutting, scope handling | 90 sec. |
| 5. Cutting a circle | The goal is to cut a circle within the limits indicated by two black lines using one of the instruments (right or left according to the examinee’s choice). | Cutting | 260 sec. |
| 6. Scope 30° | Examinees are presented with 10 boxes. For each box, they must first reveal a number on the box by touching the box with the grasper (controlled by either the right or left instrument, according to the examinee’s choice). They must then focus on the number using the 30° scope controlled by the other instrument. To successfully focus on each number, the orientation of the scope must be adjusted. | Scope handling | 140 sec. |
| 7. Needle transfer | Examinees are presented with a needles and a membrane. They must grasp the needle using one of the instruments (right or left according to the examinee’s choice), insert it through the membrane, and then use the other grasper (controlled by the other instrument) to pull the needle from the other side of the membrane. This process must be repeated six times to complete the task. | Using a needle | 150 sec. |
| 8. Needle rotation^a^ | Examinees are presented with a needles and a membrane (as in Task 10). Again, they must grasp the needle using one of the instruments (right or left according to the examinee’s choice), insert it through the membrane, and then use the other grasper (controlled by the other instrument) to pull the needle from the other side of the membrane. This process must be repeated six times, with the angle of the membrane rotating in a different direction each time. | Using a needle | 230 sec. |

^a^ This task appeared only in the initial version of the test. It was omitted from later versions based on the feedback of the participants in Phase 2.

**Figure A1**. Distributions of Performance Parameters in the Revised Test, by Task (Phase 3)


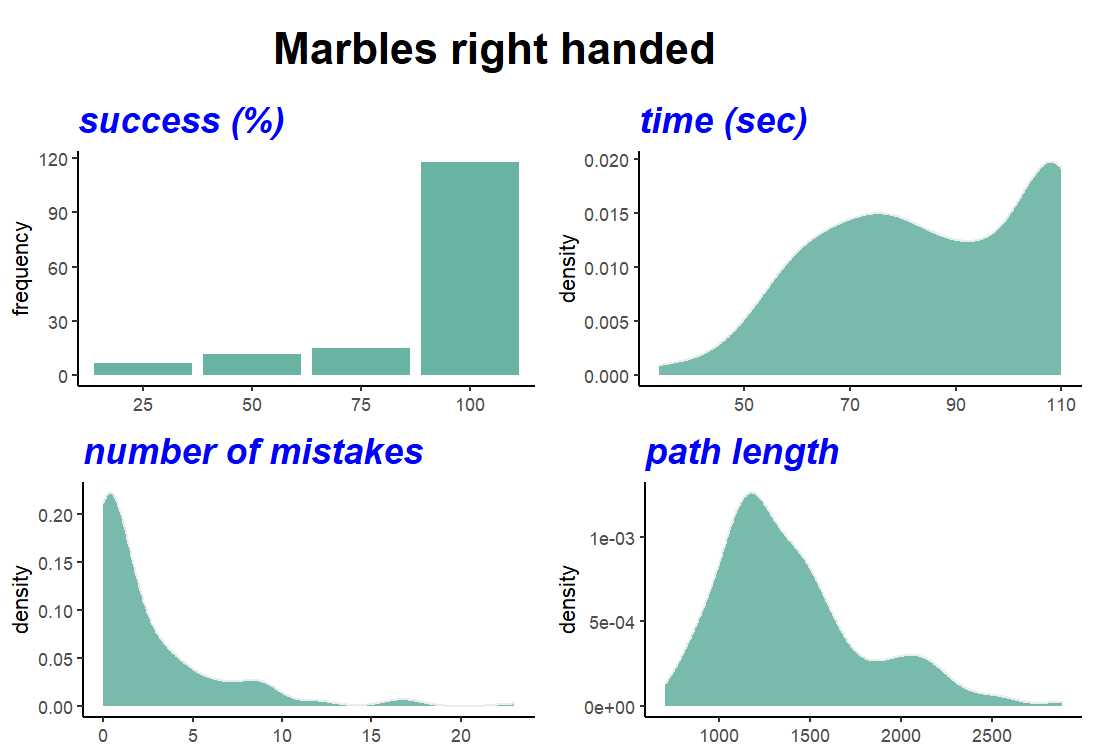

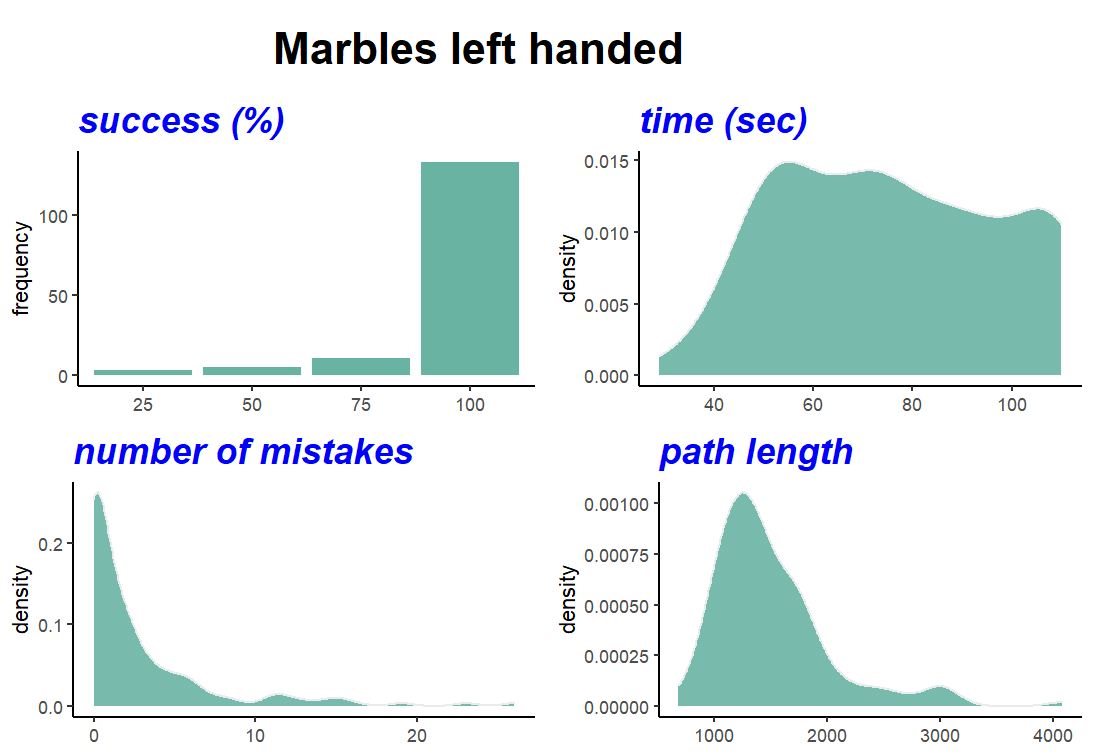


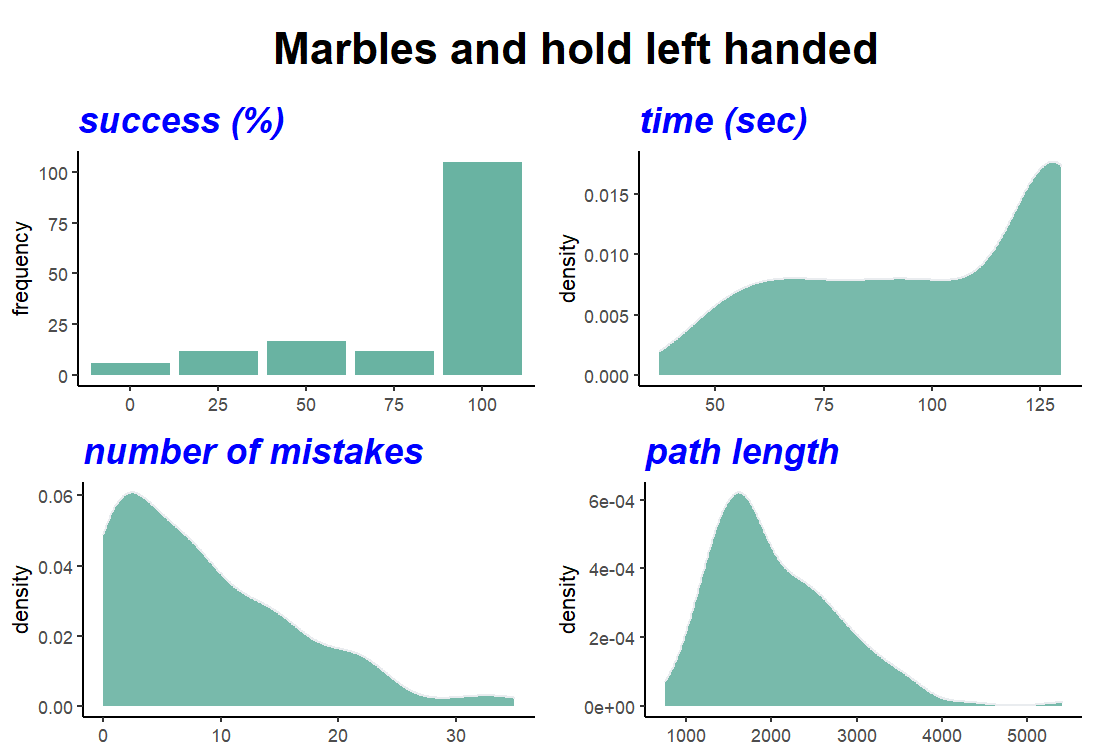

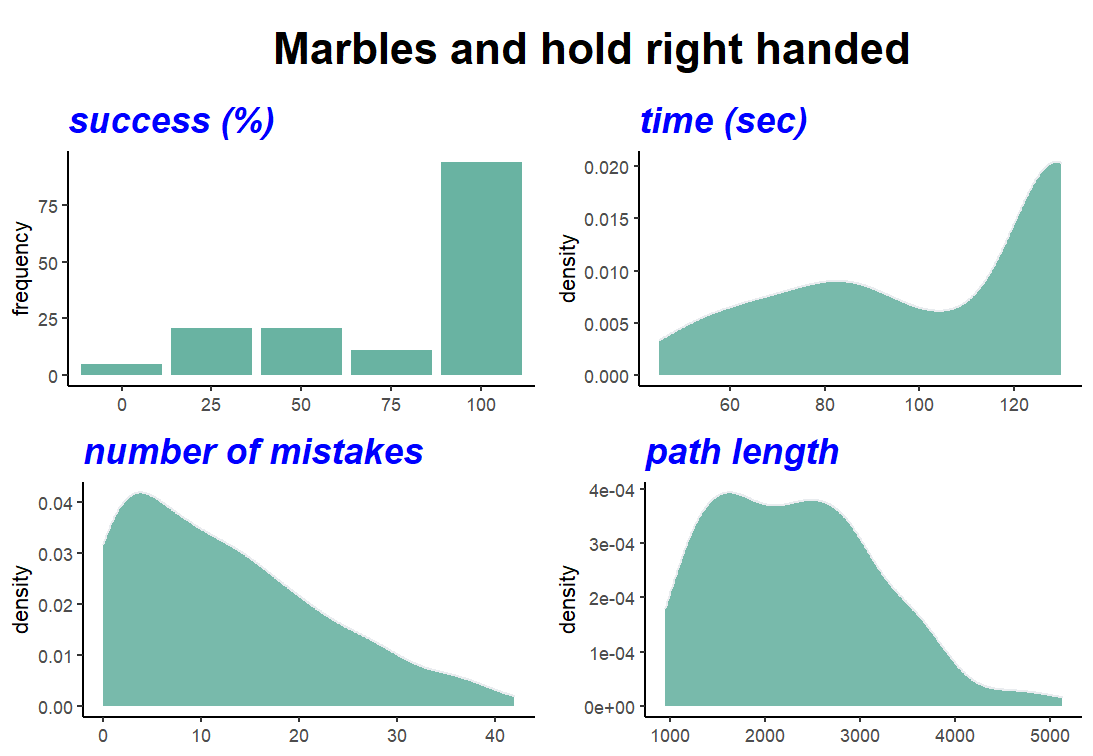


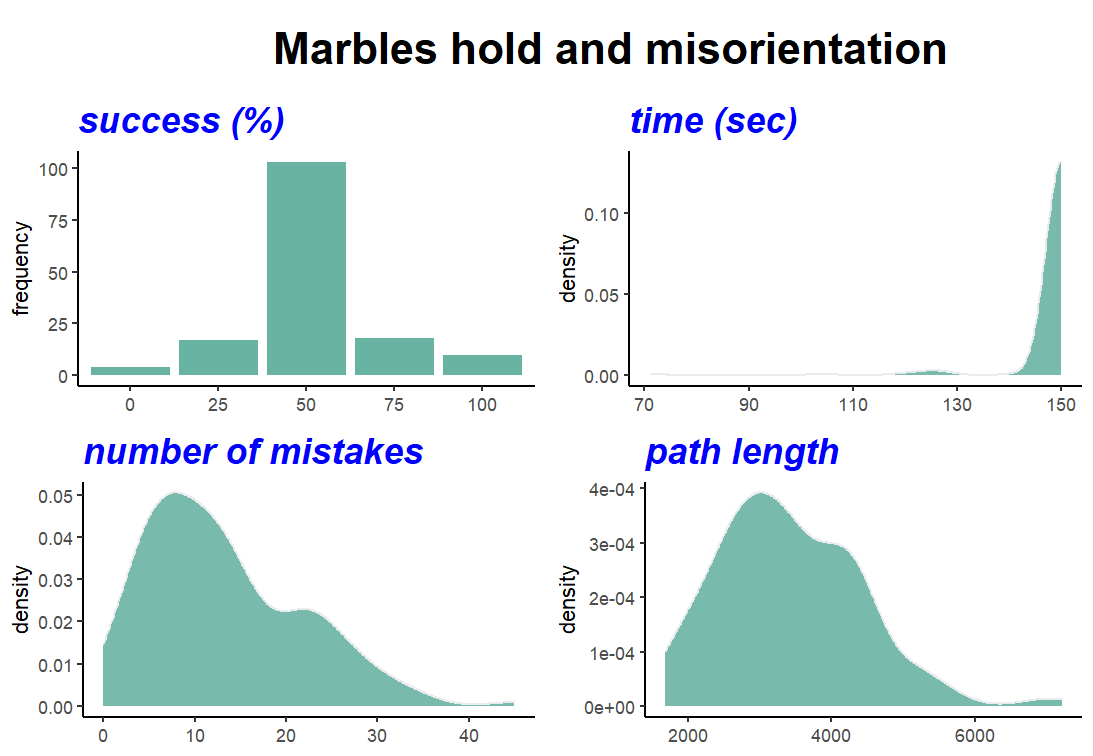


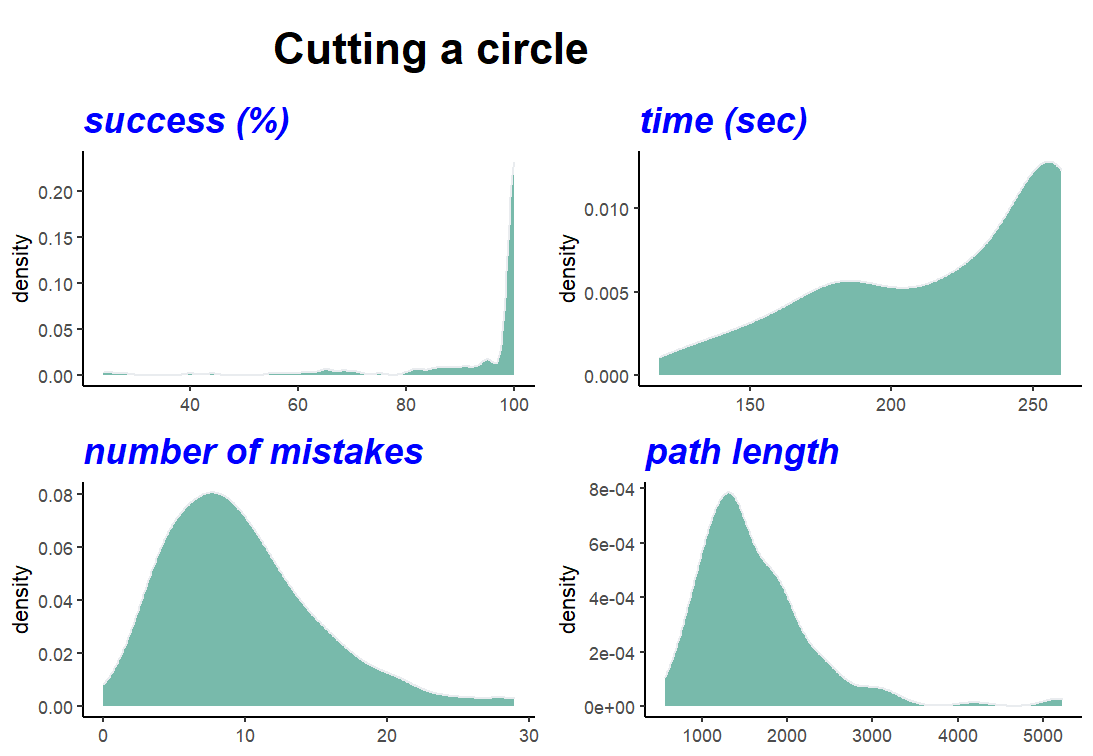

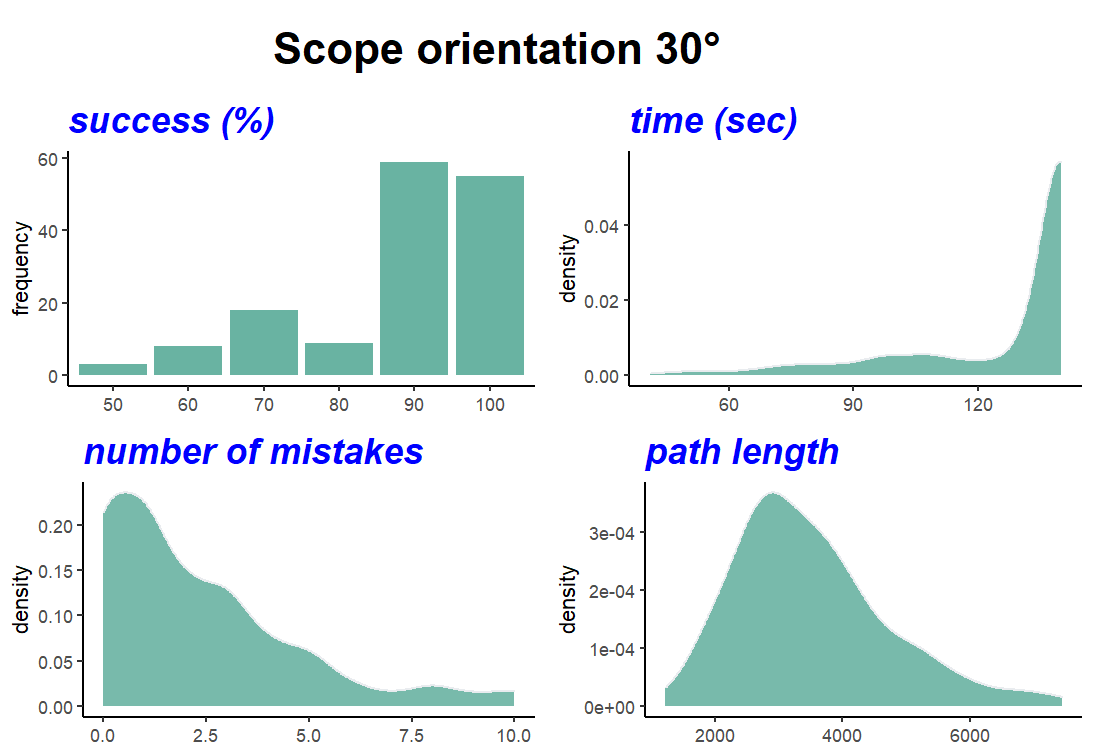

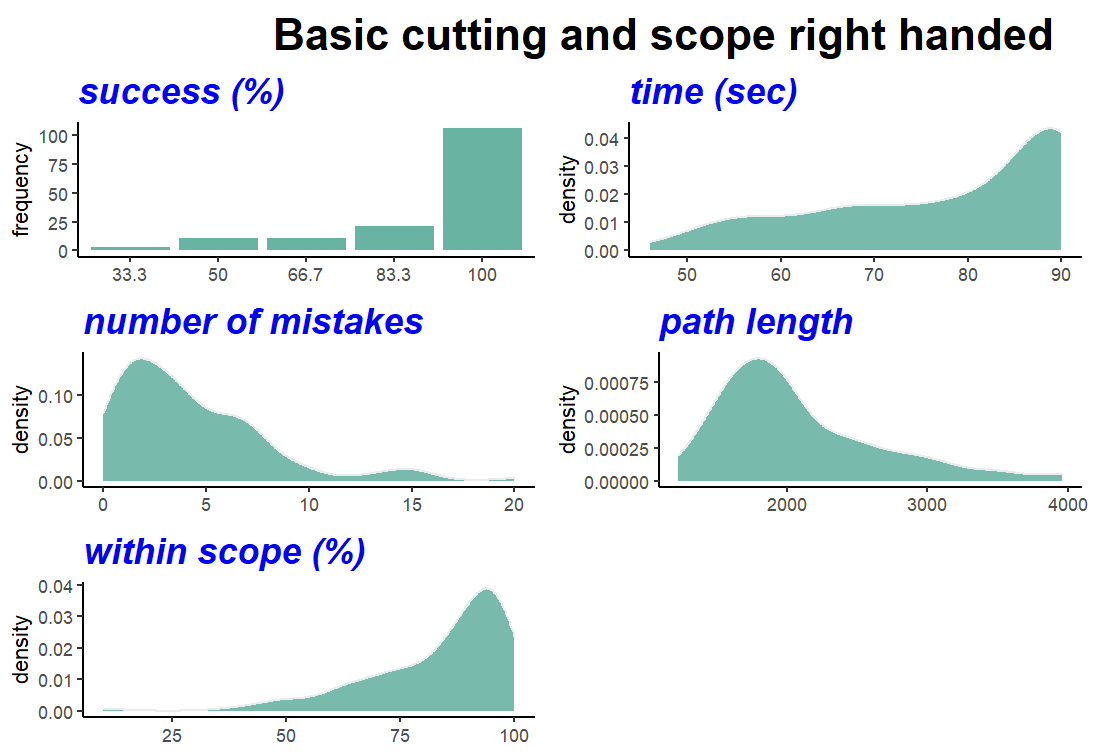

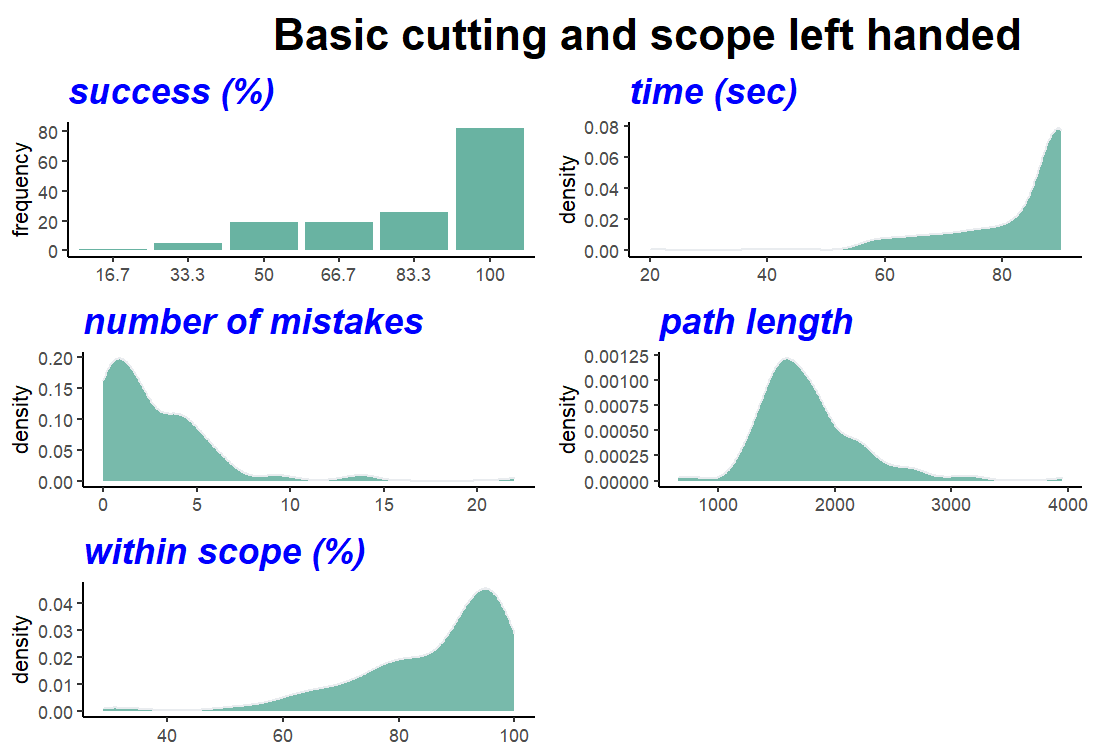


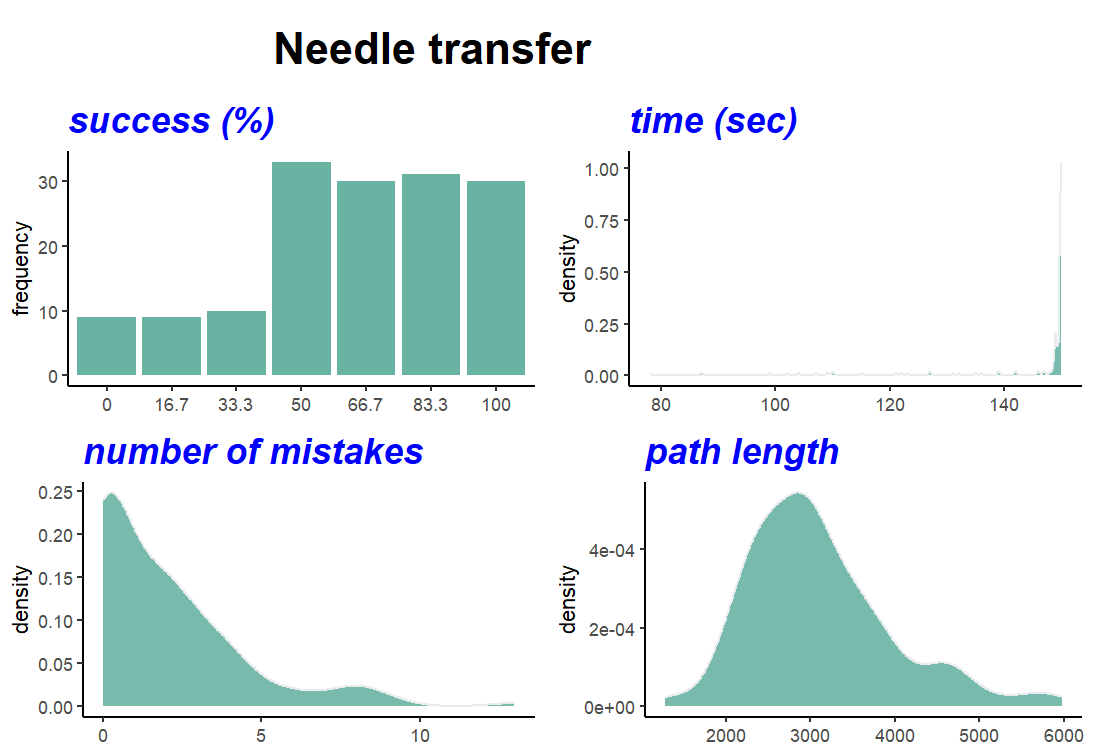


**Table A2**. Correlations Between Performance Parameters in Each Task (Phase 3)

| **Marbles (right hand)** | | | | |
| --- | --- | --- | --- | --- |
|  | *Success (%)* | *Time* | *Mistakes* | *Path length* |
| *Success (%)* |  |  |  |  |
| *Time* | -0.58^***^ |  |  |  |
| *Mistakes* | -0.57^***^ | 0.38^***^ |  |  |
| *Path length* | -0.20^*^ | 0.39^***^ | 0.57^***^ |  |
| *Based on Pearson correlations with listwise deletion.* | | | | |
| **Marbles (left hand)** | | | | |
|  | *Success (%)* | *Time* | *Mistakes* | *Path length* |
| *Success (%)* |  |  |  |  |
| *Time* | -0.52^***^ |  |  |  |
| *Mistakes* | -0.44^***^ | 0.54^***^ |  |  |
| *Path length* | -0.39^***^ | 0.62^***^ | 0.73^***^ |  |
| *Based on Pearson correlations with listwise deletion.* | | | | |

| **Marbles and hold (right hand)** | | | | |
| --- | --- | --- | --- | --- |
|  | *Success (%)* | *Time* | *Mistakes* | *Path length* |
| *Success (%)* |  |  |  |  |
| *Time* | -0.66^***^ |  |  |  |
| *Mistakes* | -0.61^***^ | 0.71^***^ |  |  |
| *Path length* | -0.54^***^ | 0.76^***^ | 0.78^***^ |  |
| *Based on Pearson correlations with listwise deletion.* | | | | |

| **Marbles and hold (left hand)** | | | | |
| --- | --- | --- | --- | --- |
|  | *Success (%)* | *Time* | *Mistakes* | *Path length* |
| *Success (%)* |  |  |  |  |
| *Time* | -0.59^***^ |  |  |  |
| *Mistakes* | -0.51^***^ | 0.63^***^ |  |  |
| *Path length* | -0.39^***^ | 0.64^***^ | 0.70^***^ |  |
| *Based on Pearson correlations with listwise deletion.* | | | | |
| **Marbles and hold – misorientation** | | | | |
|  | *Success (%)* | *Time* | *Mistakes* | *Path length* |
| *Success (%)* |  |  |  |  |
| *Time* | -0.50^***^ |  |  |  |
| *Mistakes* | -0.44^***^ | 0.21^*^ |  |  |
| *Path length* | -0.14 | 0.22^**^ | 0.39^***^ |  |
| *Based on Pearson correlations with listwise deletion.* | | | | |

| **Cutting and scope (right hand)** | | | | | |
| --- | --- | --- | --- | --- | --- |
|  | *Success (%)* | *Time* | *Mistakes* | *Path length* | *Within scope (%)* |
| *Success (%)* |  |  |  |  |  |
| *Time* | -0.49^***^ |  |  |  |  |
| *Mistakes* | -0.12 | 0.16 |  |  |  |
| *Path length* | -0.26^**^ | 0.47^***^ | 0.64^***^ |  |  |
| *Within scope (%)* | 0.33^***^ | -0.34^***^ | -0.53^***^ | -0.68^***^ |  |
| *Based on Pearson correlations with listwise deletion* | | | | | |

| **Cutting and scope (left hand)** | | | | | |
| --- | --- | --- | --- | --- | --- |
|  | *Success (%)* | *Time* | *Mistakes* | *Path length* | *Within scope (%)* |
| *Success (%)* |  |  |  |  |  |
| *Time* | -0.48^***^ |  |  |  |  |
| *Mistakes* | -0.02 | 0.05 |  |  |  |
| *Path length* | -0.07 | 0.29^***^ | 0.44^***^ |  |  |
| *Within scope (%)* | 0.28^***^ | -0.14 | -0.41^***^ | -0.65^***^ |  |
| *Based on Pearson correlations with listwise deletion.* | | | | | |

| **Cutting a circle** | | | | |
| --- | --- | --- | --- | --- |
|  | *Success (%)* | *Time* | *Mistakes* | *Path length* |
| *Success (%)* |  |  |  |  |
| *Time* | -0.45^***^ |  |  |  |
| *Mistakes* | 0.16 | 0.09 |  |  |
| *Path length* | -0.45^***^ | 0.48^***^ | 0.26^**^ |  |
| *Based on Pearson correlations with listwise deletion* | | | | |

| **Scope 30°** | | | | |
| --- | --- | --- | --- | --- |
|  | *Success (%)* | *Time* | *Mistakes* | *Path length* |
| *Success (%)* |  |  |  |  |
| *Time* | -0.51^***^ |  |  |  |
| *Mistakes* | -0.08 | 0.18^*^ |  |  |
| *Path length* | -0.23^**^ | 0.54^***^ | 0.51^***^ |  |
| *Based on Pearson correlations with listwise deletion.* | | | | |

| **Needle transfer** | | | | |
| --- | --- | --- | --- | --- |
|  | *Success (%)* | *Time* | *Mistakes* | *Path length* |
| *Success (%)* |  |  |  |  |
| *Time* | -0.46^***^ |  |  |  |
| *Mistakes* | -0.49^***^ | 0.18^*^ |  |  |
| *Path length* | -0.32^***^ | 0.31^***^ | 0.12 |  |
| *Based on Pearson correlations with listwise deletion* | | | | |

**Table A3**. Correlations Between Final Scores in Each Task (Phase 3)

|  | *Task 1* | *Task 2* | *Task 3* | *Task 4* | *Task 5* | *Task 6* | *Task 7* | *Task 8* | *Task 9* | *Task 10* |
| --- | --- | --- | --- | --- | --- | --- | --- | --- | --- | --- |
| *Task 1* |  |  |  |  |  |  |  |  |  |  |
| *Task 2* | 0.46^***^ |  |  |  |  |  |  |  |  |  |
| *Task 3* | 0.22^**^ | 0.35^***^ |  |  |  |  |  |  |  |  |
| *Task 4* | 0.39^***^ | 0.36^***^ | 0.35^***^ |  |  |  |  |  |  |  |
| *Task 5* | 0.22^**^ | 0.26^**^ | 0.30^***^ | 0.28^***^ |  |  |  |  |  |  |
| *Task 6* | 0.25^**^ | 0.28^***^ | 0.29^***^ | 0.34^***^ | 0.21^*^ |  |  |  |  |  |
| *Task 7* | 0.26^**^ | 0.22^**^ | 0.16^*^ | 0.32^***^ | 0.11 | 0.46^***^ |  |  |  |  |
| *Task 8* | 0.32^***^ | 0.34^***^ | 0.30^***^ | 0.31^***^ | 0.32^***^ | 0.39^***^ | 0.35^***^ |  |  |  |
| *Task 9* | 0.26^**^ | 0.28^***^ | 0.27^***^ | 0.35^***^ | 0.29^***^ | 0.37^***^ | 0.39^***^ | 0.36^***^ |  |  |
| *Task 10* | 0.24^**^ | 0.37^***^ | 0.36^***^ | 0.41^***^ | 0.24^**^ | 0.45^***^ | 0.41^***^ | 0.47^***^ | 0.55^***^ |  |
| *Based on Pearson correlations with listwise deletion.* | | | | | | | | | | |

**Table A4**. Item Analysis (Phase 3)

| Task | Mean^a^ | SD | Skew | Item discrimination | α if deleted |
| --- | --- | --- | --- | --- | --- |
| Marbles (right hand) | 0 | 0.77 | -1.14 | 0.46 | 0.81 |
| Marbles (left hand) | 0 | 0.81 | -1.56 | 0.52 | 0.81 |
| Marbles and hold (right hand) | 0 | 0.87 | -0.18 | 0.46 | 0.81 |
| Marbles and hold (left hand) | 0 | 0.83 | -0.48 | 0.56 | 0.80 |
| Marbles and hold – misorientation | 0 | 0.7 | 1.41 | 0.39 | 0.82 |
| Cutting and scope (right hand) | 0 | 0.72 | -0.88 | 0.53 | 0.81 |
| Cutting and scope (left hand) | 0 | 0.65 | -0.89 | 0.46 | 0.81 |
| Cutting a circle | 0 | 0.71 | 0.05 | 0.56 | 0.80 |
| Scope 30° | 0 | 0.67 | -0.81 | 0.55 | 0.80 |
| Needle transfer | 0 | 0.7 | 0.11 | 0.62 | 0.80 |
| Cronbach’s alpha = 0.83 | | | | | |

^a^ The mean score of all tasks is equal to 0 since the total score of each task was calculated as the average of the standardized parameter scores (each having a mean of 0).

**Table A5**. Summary of Paired t-tests Analyses Comparing Males and Females' Parameter Scores (Phase 3)

| Performance parameter | Males (n = 82) | | Females (n = 70) | |  |  |  | *t*(150) | *p* | Cohen's *d* |
| --- | --- | --- | --- | --- | --- | --- | --- | --- | --- | --- |
|  | *M* | *SD* | *M* | *SD* |  |  |  |  |  |  |
| Success (%) | 84.84 | 11.04 | 78.09 | 13.84 |  |  |  | 3.28* | 0.001 | 0.53 |
| Time | 114.10 | 11.94 | 119.49 | 13.05 |  |  |  | 2.64* | .009 | 0.43 |
| Mistakes | 5.69 | 2.32 | 6.44 | 3.21 |  |  |  | 1.62 | 0.11 | 0.27 |
| Path length | 2165.15 | 400.78 | 2416.74 | 569.04 |  |  |  | 3.10* | 0.002 | 0.51 |
| Within scope (%) | 85.12 | 14.18 | 84.77 | 11.46 |  |  |  | 0.17 | 0.87 | 0.02 |

**Table A6**. Summary of Hierarchal Regression Analysis (Phase 3)

| Variable | $\beta$ | *F* | *sr*^2^ | *R* | *R*^2^ | Δ*R*^2^ |
| --- | --- | --- | --- | --- | --- | --- |
| Step 1 |  |  |  | 0.25 | 0.06 | 0.06 |
| Experience with surgical simulators | 0.18 | 5.01* | 0.03 |  |  |  |
| Experience with video games | 0.14 | 3.09 | 0.02 |  |  |  |
| Step 2 |  |  |  | 033 | 0.11 | 0.05 |
| Experience with surgical simulators | 0.17 | 4.74* | 0.03 |  |  |  |
| Experience with video games | 0.02 | 0.04 | 0.00 |  |  |  |
| Gender, female | -0.25 | 7.54** | 0.06 |  |  |  |

Note. N = 152; *p < .05, **p < .01
